# Use of erythromycin and metoclopramide in hospitalized dogs: a multicenter historical cohort study

**DOI:** 10.3389/fvets.2025.1551312

**Published:** 2025-04-25

**Authors:** Ee Fung Teo, Claire R. Sharp, Corrin J. Boyd, Weiqin Chee

**Affiliations:** ^1^Western Australian Veterinary Emergency and Specialty (WAVES), Success, WA, Australia; ^2^School of Veterinary Medicine, Murdoch University, Murdoch, WA, Australia; ^3^Centre for Terrestrial Ecosystem Science and Sustainability, Harry Butler Institute, Murdoch University, Murdoch, WA, Australia

**Keywords:** prokinetic, ileus, GI dysmotility, gastroparesis, motilin receptor

## Abstract

**Introduction:**

Prokinetics are used to treat gastrointestinal (GI) dysmotility in critically ill dogs but there have been no published studies characterizing their use. The objective of this multi-institutional retrospective cohort study was to describe the use of the prokinetics erythromycin and metoclopramide in dogs hospitalized in two institutions. We hypothesized that there would be change over time and differences between institutions in the use of erythromycin and metoclopramide.

**Methods:**

Dogs for inclusion were identified by fee code searches for injectable erythromycin and metoclopramide in the electronic medical record systems of The Animal Hospital at Murdoch University and Western Australian Veterinary Emergency and Specialty Hospital for the years 2018 and 2023. 75 cases from each hospital in each year were selected for inclusion from the search results using a formal randomization procedure to yield a total case number of 300. Data collected for each dog included signalment, diagnosis, reason(s) for starting prokinetics, the injectable prokinetic(s) used, frequency, and doses. Chi square or Fisher’s exact tests were used as appropriate to compare the proportions of dogs receiving metoclopramide alone, erythromycin alone, or both prokinetics in 2018 and 2023, the proportions of dogs receiving metoclopramide or erythromycin as sole prokinetics between the two institutions, and the proportions of dogs receiving a single prokinetic versus dual prokinetics between the two institutions.

**Results:**

Primary GI diseases accounted for the majority of the diagnoses. The most common reasons for starting a prokinetic were vomiting, an imaging diagnosis of ileus, prophylaxis following abdominal surgery, and regurgitation. Metoclopramide was administered as a sole prokinetic in the majority of dogs, fewer cases received erythromycin alone, or both prokinetics. Use of metoclopramide alone decreased from 2018 to 2023, with more dogs receiving erythromycin alone or both prokinetics in 2023. There were also significant differences in prokinetic use between institutions.

**Discussion:**

Prospective studies to investigate the effectiveness and safety of metoclopramide and erythromycin as prokinetics in dogs are warranted.

## 1 Introduction

Gastrointestinal (GI) dysmotility is common in critically ill dogs (1). Gastrointestinal dysmotility increases the risk of regurgitation and vomiting, which predisposes to aspiration events that can lead to bacterial pneumonia and/or chemical pneumonitis (2); other complications include fluid sequestration, delayed nutrient delivery, and bacterial translocation (1). In human beings, GI dysmotility has been associated with increased length of hospitalization and mortality in a variety of diseases (3, 4). Despite being hospitalized for similar diseases, the prevalence and consequences of GI dysmotility have not been well characterized in hospitalized dogs (5).

Medications with a prokinetic effect on the GI tract, subsequently referred to as prokinetics, are commonly prescribed to treat GI dysmotility. Prokinetics aid in the amplification and coordination of GI muscular contractions to facilitate the aboral transit of luminal contents (6). Metoclopramide and erythromycin are frequently used prokinetics in dogs (1). Metoclopramide is a dopaminergic (D_2_) receptor antagonist, 5-HT_3_ antagonist, and 5-HT_4_ agonist (7). Dopaminergic antagonism in the myenteric plexus promotes the release of acetylcholine. Muscarinic stimulation by acetylcholine in turn increases lower esophageal sphincter and gastric tone, intragastric pressure, antroduodenal coordination, and rate of gastric emptying (8, 9). In addition, metoclopramide is antiemetic, making it an attractive first choice drug for concurrent vomiting and GI dysmotility (1). Erythromycin is a macrolide antibiotic that increases GI motility through species dependent mechanisms. In dogs, mechanisms may include motilin receptor stimulation, increased release of endogenous motilin, and 5-HT_3_-cholinergic pathways (10–13). Erythromycin induces strong contractions in the gastric body and antrum, duodenum, and jejunum of dogs, mimicking naturally occurring interdigestive migrating contractions in the GI tract (10).

A recent scoping review of prokinetic use in hospitalized adult human patients identified 102 studies on the topic, encompassing 8,830 patients (14). This included 68 clinical trials of which 60% were conducted in an ICU setting, with feeding intolerance the main indication. Across all studies metoclopramide was used most commonly (49%), followed by erythromycin (31%), and less commonly cisapride and other agents (14). However, the authors reported that patient-centered outcomes were rarely assessed, and the quality of evidence was low, thus they could not draw firm conclusions regarding the balance between desirable and undesirable effects of prokinetics (14). Despite this, guidelines of the European Society for Clinical Nutrition and Metabolism (ESPN) recommend intravenous erythromycin as the first line prokinetic in critically ill patients with gastric feeding intolerance with a strong consensus, or alternatively suggest intravenous metoclopramide or a combination of metoclopramide and erythromycin for prokinetic therapy (15, 16). Cisapride is not mentioned in the ESPN guidelines, as despite evidence of efficacy, it has been withdrawn from the market in many parts of the world due to the risk of cardiac arrhythmias (17). The ESPN recommendations were based on their own meta-analysis that demonstrated that feeding intolerance was improved with the use of prokinetics, particularly erythromycin, but did not demonstrate a difference in the outcome of pneumonia (15).

In contrast, current literature regarding prokinetics in dogs is limited to experimental studies in healthy dogs and recommendations in review papers and book chapters. To the authors’ knowledge there are no published studies characterizing the clinical use of erythromycin and metoclopramide in dogs. It is the authors’ anecdotal impression that the use of erythromycin as a prokinetic in dogs has increased within the last decade, albeit with variation among clinicians.

The objective of this study was to describe the use of the prokinetics erythromycin and metoclopramide in hospitalized dogs. We hypothesized that there would be change over time and differences between institutions in the use of erythromycin and metoclopramide.

## 2 Materials and methods

This was a multicenter retrospective cohort study. Dogs for inclusion were identified by fee code searches for the specific billing items for injectable erythromycin and metoclopramide in the electronic medical record systems from The Animal Hospital at Murdoch University (TAHMU) and Western Australian Veterinary Emergency and Specialty Hospital (WAVES) for the years 2018 and 2023 (January 1 to December 31, for each year). These are primary accession and referral hospitals in metropolitan Perth, Western Australia. The year 2018 was chosen as injectable erythromycin was first available at both hospitals in this year, and the year 2023 was subsequently chosen to capture dose changes that may have occurred over the five-year intervening period.

With the expectation that the fee code search would yield a large number of cases, a sample size calculation was performed to guide the minimum case number for inclusion. A two-sided test of proportions determined that a sample size of at least 55 per group would be needed to detect an increase in the proportion of dogs receiving erythromycin in addition to metoclopramide, compared to metoclopramide alone, from 25% to 50% with an alpha of 0.05 and power of 0.8. These proportions were based on anecdotal impression of a substantial increase in erythromycin usage, rather than objective data. As such, a slightly larger sample size of 75 cases per year, per institution was chosen.

Cases for inclusion were selected from the fee code search results using a formal randomization procedure (Supplementary Methods). The randomized cases were then sequentially screened for inclusion by one of the authors until the desired number of cases in each year at each institution was reached.

Cases were excluded from the study if there were duplicate entries for the same hospitalization or if their medical records were incomplete relative to the required study data collection parameters. Dogs that received only outpatient treatment, a single dose of metoclopramide after induction of emesis, where prokinetics were billed but not administered, or if hospitalization extended beyond the years 2018 and 2023, were excluded.

Dog data was extracted from the medical records and recorded in an online data collection form in REDCap (Research Electronic Data Capture) (Supplementary Data Collection Instrument) (18, 19). Data collected for each dog included signalment (calculated age, sex, breed), body weight, and clinician determined diagnosis (Supplementary Data Sheet). The primary service managing the dog when the first prokinetic was prescribed was recorded as either the emergency and critical care, surgery, or internal medicine services.

The reason(s) for starting prokinetics, injectable prokinetic(s) used, order, frequency, initial and maximum daily doses, and duration of injectable prokinetics, as well as whether oral prokinetics were prescribed was documented. The reason for starting prokinetics was categorized based on *a priori* criteria, notably clinical signs (vomiting and/or regurgitation), an imaging diagnosis of ileus, intolerance of enteral feeding, prophylactically for a variety of conditions [e.g., brachycephalic obstructive airway syndrome (BOAS) (20), enteral feeding, post-abdominal surgery], BOAS with a history of regurgitation, or unknown. The term ileus was used to refer to both decreased motility of the stomach (also known as gastroparesis), and the small intestine. An imaging diagnosis of ileus was based on documentation in the diagnostic imaging report, using terms such as “ileus,” “lack of GI motility,” or “absence of GI contractions.” We included both point-of-care ultrasound (POCUS) performed by an emergency or critical care clinician, and formal abdominal ultrasound performed by a board-certified radiologist, or radiology resident under supervision. Additionally, potential risk factors for the development of ileus were recorded based on an *a priori* list (Supplementary Data Collection Instrument). Placement of a feeding tube, type of tube, and whether gastric residual volumes were measured, was recorded.

Presence of GI clinical signs other than vomiting or regurgitation (inappetence, diarrhea and/or abdominal pain), as well as the use of other GI medications, was documented. Information was collected about progression of diarrhea, if present prior to commencement of prokinetics, or whether new diarrhea developed after prokinetics were commenced. These data were collected given concern that prokinetics may precipitate diarrhea (21). A free text field in the data collection instrument allowed description of any side effects of prokinetic use that were documented in the medical record. Although this study was not designed to investigate the potential for the development of antimicrobial resistance secondary to erythromycin use, data pertaining to bacterial cultures performed within 3 months of erythromycin administration was collected. Given that vomiting and regurgitation can predispose to aspiration pneumonia and/or pneumonitis, a clinical diagnosis of aspiration pneumonia was noted if documented in the medical record, as well if aspiration pneumonia was confirmed by cytology and/or culture of bronchoalveolar lavage fluid.

Outcome of hospitalization was recorded as discharged, euthanized, or died naturally. Among the dogs discharged, differentiation was made regarding the nature of the discharge (i.e., to another veterinarian, home, against medical advice, or for imminent euthanasia).

### 2.1 Statistical methods

Descriptive statistics were generated within REDCap. Continuous variables were expressed as median (Min–Max, Q1–Q3). Categorical data were presented as counts or proportions (percentages). Statistical comparisons were performed in R (22). The proportions of dogs receiving metoclopramide alone, erythromycin alone, or both prokinetics were compared between 2018 and 2023 with a Chi square test. The proportions of dogs receiving metoclopramide and erythromycin as sole prokinetics, were compared between the two institutions with a Fisher’s exact test. Additionally, the proportions of dogs receiving a single prokinetic vs. dual prokinetics were compared between the two institutions with a Chi square test. A *p* value of <0.05 was considered significant.

## 3 Results

**Figure 1:**
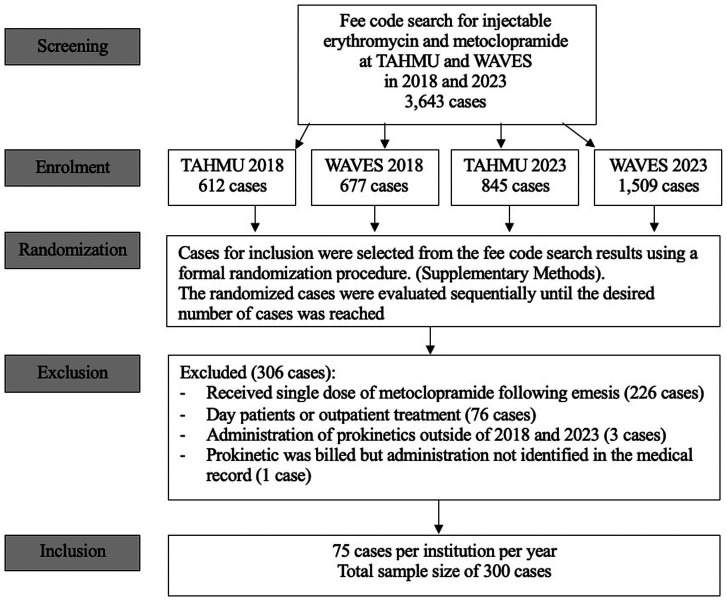
CONSORT diagram of screening, randomization, and exclusion of cases.

The fee code search yielded 3,643 unique dog cases that received metoclopramide and/ or erythromycin. This included 1,289 dogs in 2018 (677 at WAVES, 612 at TAHMU) and 2,354 dogs in 2023 (1,509 at WAVES and 845 at TAHMU). During sequential screening of the randomized cases, 306 dogs were excluded, and case inclusion stopped as planned once 75 cases per year per institution fulfilling inclusion criteria were identified (Figure 1). The majority of the excluded cases received a single dose of metoclopramide following induction of emesis (*n* = 226, 73.8%) (Figure 1).

Median age of dogs was 60 months (Min–Max 0.1–214, Q1–Q3 22.8–102.8). Male neutered (125, 41.7%) and female spayed dogs (115, 38.3%) were represented most commonly, followed by male entire (38, 12.7%) and female entire (22, 7.3%) dogs. Mixed breed dogs were most common (90, 30%). Dog breeds represented by 10 or more cases include French bulldog (26, 8.7%), Labrador retriever (26, 8.7%), Staffordshire bull terrier (18, 6%), golden retriever (13, 4.3%), and Border collie (12, 4%). Forty-one additional breeds were represented by 10 dogs or fewer. The median body weight was 16.7 kg (Min–Max 0.98–58, Q1–Q3 9.4–28.5).

Primary GI diseases accounted for the majority of the diagnoses, the single most common condition being acute vomiting and/or diarrhea with no definitive diagnosis (69, 23%), followed by a GI foreign body (55, 18.3%) that was either non-obstructive and managed medically, or obstructive and managed surgically with post-operative prokinetic use. Other primary GI diagnoses included inflammatory GI disease (14, 4.7%), infectious GI disease (9, 3%), hiatal hernia (9, 3%), food engorgement (6, 2%), GI neoplasia (6, 2%), gastric dilatation and volvulus (4, 1.3%), esophagitis (3, 1%), and one case each of gastroesophageal reflux disease, megaesophagus, mesenteric volvulus, colonic torsion, and sepsis of GI origin. A proportion of dogs had more than one diagnosis.

Extra GI diagnoses included non-GI neoplasia (26, 8.7%), BOAS (21, 7.0%), pancreatitis (17, 5.7%), liver disease (15, 5%), respiratory disease (15, 5%), toxicoses (9, 3.0%), trauma (7, 2.4%), genitourinary disease (6, 2%), sepsis of non-GI origin (5, 1.7%), neurologic disease (4, 1.3%), kidney disease (3, 1.0%), endocrine disorders (hypoadrenocorticism, hypothyroidism) (3, 1.0%), anaphylaxis (3, 1.0%), immune mediated hemolytic anemia (3, 1.0%), and elapid snake envenomation (2, 0.7%). There was one case each of septic peritonitis, tracheal foreign body, retrobulbar abscess, thermal burns, left sided congestive heart failure, and vestibular disease secondary to otitis media. In five cases (1.7%) the diagnosis was unclear from the medical record.

The most common reasons for starting a prokinetic were vomiting (203, 67.7%), an imaging diagnosis of ileus (117, 39%), prophylaxis following abdominal surgery (63, 21%) and regurgitation (47, 15.7%). Other reasons included prophylaxis for dogs with BOAS with a history of regurgitation (20, 6.7%), or without history of regurgitation (18, 6%), and prophylaxis for enteral feeding (20, 6.7%). In 12 cases (4%) the reason for starting a prokinetic was unclear from the medical record. A proportion of dogs had more than one reason for starting prokinetics. Other clinical signs and physical examination findings included abdominal pain (94, 31.3%), diarrhea (79, 26.3%) and inappetence (60, 20%). The most frequent prescribing service of the first prokinetic was emergency and critical care (267, 90.5%), followed by surgery (22, 7.5%) and internal medicine (6, 2%).

Risk factors for ileus included dehydration (228, 76.0%), opioids (160, 53.3%), hypokalemia (143, 47.7%), general anesthesia (131, 43.7%), hypovolemia (106, 35.3%), abdominal surgery (70, 23.3%), hypoxia (30, 10.0%), hypotension during general anesthesia (26, 8.7%), sepsis (20, 6.7%), vasopressor(s) (19, 6.3%), other electrolyte abnormalities (11, 3.7%), mechanical ventilation in the intensive care unit (7, 2.3%), and non-infectious systemic inflammatory response syndrome (5, 1.7%). Feeding tubes were placed in 58 dogs which included nasogastric tubes (47, 15.7%), esophagostomy tubes (5, 1.7%), or percutaneously or surgically placed gastrostomy tubes (7, 2.3%). Of these, 41 dogs (75.9%) had measurements of gastric residual volumes documented.

Table 1 displays the number and proportions of cases receiving metoclopramide alone, erythromycin alone, or both, at each hospital in each year. Overall, metoclopramide was administered as a sole prokinetic in the majority of dogs (160/300, 53.3%); fewer cases received erythromycin alone (46, 15.3%), or both prokinetics (94, 31.3%). Of the cases receiving both prokinetics, metoclopramide was most frequently commenced first (43, 45.7%), followed by both drugs being prescribed at the same time (37, 39.4%), with only a small number of cases receiving erythromycin first (11, 11.7%). In three cases (3.2%) the order of commencement of prokinetics was unable to be discerned.

**Table 1:** Summary of prokinetics used in dogs treated at two referral hospitals in 2018 and 2023 (displayed as number, and percentage).

Prokinetics used	The Animal Hospital at Murdoch University (TAHMU)		Western Australian Veterinary Emergency and Specialty Hospital (WAVES)	
	2018	2023	2018	2023
Metoclopramide alone	28 (37.33%)	9 (12%)	70 (93.33%)	53 (70.67%)
Erythromycin alone	13 (17.33%)	33 (44%)	0	0
Both prokinetics	34 (45.33%)	33 (44%)	5 (6.67%)	22 (29.33%)

75 dogs were included from each hospital from each year.

Use of metoclopramide alone decreased from 2018 to 2023, with more dogs receiving erythromycin alone or both prokinetics in 2023 (Table 2, *p* < 0.001). There were also significant differences in prokinetic use between institutions; TAHMU cases were more likely to be prescribed erythromycin as a sole prokinetic, or receive dual prokinetics, than cases at WAVES, where the majority received metoclopramide alone (Tables 3, 4, *p* < 0.001 for both comparisons).

**Table 2:** Numbers of dogs receiving metoclopramide alone, erythromycin alone, or both, in the years 2018 and 2023, with data from both The Animal Hospital at Murdoch University and Western Australian Veterinary Emergency and Specialty combined.

Year	Metoclopramide only	Erythromycin only	Metoclopramide and erythromycin	Row totals
2018	98	13	39	150
2023	62	33	55	150
Column totals	160	46	94	300 (Grand total)

The Chi square statistic is 19.5191. The *p* value is 0.000058. The result is significant at *p* < 0.05.

**Table 3:** Numbers of dogs receiving metoclopramide and erythromycin as sole prokinetics at The Animal Hospital at Murdoch University (TAHMU) and Western Australian Veterinary Emergency and Specialty (WAVES), with data from 2018 and 2023 combined.

Institution	Metoclopramide only	Erythromycin only	Row totals
TAHMU	37	46	83
WAVES	123	0	123
Column totals	160	46	206

The Fisher exact test statistic value is <0.00001. The results is significant at *p* < 0.05.

**Table 4:** Numbers of dogs receiving a sole prokinetic vs. dual prokinetics at The Animal Hospital at Murdoch University (TAHMU) and Western Australian Veterinary Emergency and Specialty (WAVES), with data from 2018 and 2023 combined.

Institution	Sole prokinetic	Dual prokinetics	Row totals
TAHMU	83	67	150
WAVES	123	27	150
Column totals	206	94	300

The Fisher exact test statistic value is <0.00001. The result is significant at *p* < 0.05.

Metoclopramide was most frequently administered as an IV CRI after an initial bolus (139/254, 54.7%), with other dosing approaches including IV CRI without an initial bolus (84, 33.1%), IV every 8 h (15, 5.9%), IV once only (14, 5.5%) and IV every 6 h (4, 1.6%). The median initial daily dose administered was 2 mg/kg (Min–Max 0.3–3, Q1–Q3 2–2), and the median maximum daily dose was 2 mg/kg (Min–Max 0.3–3, Q1–Q3 2–2). In the 254 cases where injectable metoclopramide was prescribed, the duration of therapy was less than or equal to 24 h (102, 40.2%), 24 to 48 h (82, 32.3%), 48 to 72 h (39, 15.4%), and more than 72 h (31, 12.2%). Eighty-six dogs were continued on oral metoclopramide following their IV course.

Erythromycin was most frequently administered IV every 8 h (130/140, 92.9%), followed by IV every 6 h (5, 3.6%) and IV once only (4, 2.9%). The median initial daily dose administered was 3 mg/kg (Min–Max 0.9–9, Q1–Q3 3–3), and the median maximum daily dose was 3 mg/kg (Min–Max 1–9, Q1–Q3 3–3). In the 140 cases where injectable erythromycin was prescribed, the duration of therapy was less than or equal to 24 h (53, 37.9%), 24 to 48 h (51, 36.4%), 48 to 72 h (18, 12.9%), and more than 72 h (18, 12.9%). Forty-five dogs were continued on oral erythromycin following their IV course.

There were no documented side effects attributed to the administration of either prokinetic in the medical records of the included dogs. In the 79 dogs that had diarrhea prior to prokinetics, the diarrhea improved in 45 (57.0%), stayed the same in 24 (30.4%), and worsened in 7 (8.9%). In 3 cases (3.8%) it was unclear if there was a change in the diarrhea after starting a prokinetic. New diarrhea developed in an additional 13 cases after starting prokinetics.

Seven dogs that received erythromycin subsequently had bacterial cultures performed, which included urine, transtracheal wash, and abdominal fluid. Three of these cultures were positive. One grew *Staphylococcus pseudintermedius* susceptible to erythromycin and clindamycin. Susceptibility to erythromycin and clindamycin was not reported for the other isolates (*Enterococcus* spp., non-hemolytic *Escherichia coli*, *Pasteurella dagmatis*, and *Streptococcus canis*).

Only a small number of dogs were documented to have aspiration pneumonia; 21 dogs before prokinetic administration, and 8 dogs after prokinetic administration. None of these dogs had a bronchoalveolar lavage for cytology and culture performed.

Many dogs also received other GI medications including maropitant (222, 74%), proton pump inhibitors (147, 49%), ondansetron (120, 40%), probiotics (20, 6.7%), sucralfate (13, 4.3%), cisapride (8, 2.7%), bismuth subsalicylate (4, 1.3%), and barium (for the purpose of treating GI ulcers) (1, 0.3%).

The majority of dogs survived to discharge home (260, 86.7%). Smaller numbers of dogs were euthanized (22, 7.3%), discharged for imminent euthanasia (10, 3.3%), or discharged to another veterinarian (8, 2.7%).

## 4 Discussion

This study of the prokinetic medications metoclopramide and erythromycin in hospitalized dogs supported our hypotheses by demonstrating a difference in the use of these medications between the two institutions and increased use of erythromycin and dual prokinetics in 2023 compared to 2018. The substantial number of cases retrieved by the search strategy in this study highlights the frequent use of these prokinetics in these institutions. Our data also suggests that GI dysmotility was frequently diagnosed in dogs at these hospitals, but prospective research is required to definitively determine the prevalence of this condition in hospitalized dogs. Dogs in this study generally had a good outcome with 85.7% surviving to discharge, but the effect of GI dysmotility and its treatment with prokinetic therapy on outcome is unknown.

The institutional differences identified in this study are on the one-hand not surprising given lack of institutional protocols surrounding prokinetic use in the study hospitals, and lack of evidence to support one prokinetic over the other, both in terms of effectiveness and safety, in dogs. While there is evidence of the efficacy of each drug individually in experimental studies in dogs (7, 10–12), comparative clinical data is lacking, and so clinician perceptions of effectiveness may influence prescribing. It is possible that clinical studies supporting the concurrent use of metoclopramide and erythromycin in human beings have led clinicians to use both drugs in dogs (23–25). Different clinicians may also weigh up the potential for adverse effects differently. For example, concern for antimicrobial resistance or effects on the microbiome with erythromycin use may prompt a clinician to avoid its use. Conversely, concerns for the extrapyramidal effects or drug interactions of metoclopramide may prompt a clinician to use erythromycin first. Ultimately, prospective clinical studies investigating comparative effectiveness and safety are needed to facilitate more informed clinical decision making regarding the use of prokinetics in hospitalized dogs.

On the other hand, the institutional differences are interesting given that the two studied hospitals are in the same geographic area with many veterinarians having studied and/or done their specialty training at the same local veterinary school. Indeed, both hospitals have a similar structure with case management by emergency clinicians, as well as specialist criticalists, internists, surgeons, and their residents. Nonetheless, factors influencing individual clinician prescribing of prokinetics were beyond the scope of this study. Given the similarities between the two study hospitals it may be that variation in prokinetic use is even greater in the broader veterinary community than was evidenced in this study.

Despite the differences between institutions, both institutions saw an increase in the proportion of erythromycin used in 2023, when compared to 2018. The emergence of erythromycin use might indicate that clinicians feel that metoclopramide alone was insufficient to treat GI dysmotility. While our study was not designed to assess effectiveness, increasing use suggests that anecdotally clinicians may appreciate some advantage of erythromycin, highlighting the need for prospective research to assess effectiveness.

The dosing regimens reported in this study were mostly consistent with the current recommendations for each medication in veterinary formularies, of 0.08–0.16 mg/kg/h (2–4 mg/kg/day) for metoclopramide, and 0.5–1 mg/kg IV every 8 h for erythromycin (26). In human studies, both metoclopramide and erythromycin are associated with the rapid development of tachyphylaxis which leads to a reduction in efficacy of prokinetic therapy (27, 28). The use of combination therapy with metoclopramide and erythromycin is associated with improved effectiveness in multiple studies (23–25), possibly due to a lesser degree of drug tachyphylaxis (23). Prospective studies are warranted to investigate whether tachyphylaxis develops with the use of metoclopramide and erythromycin in hospitalized dogs.

The most common reason for commencing prokinetics in this study was vomiting. Metoclopramide serves a dual purpose for vomiting as it acts on the chemoreceptor trigger zone via dopamine antagonism to cause central antiemetic effects and peripherally via 5-HT_3_ serotonergic antagonism and 5-HT_4_ serotonergic agonism that increases gastroesophageal sphincter tone and gastric emptying (9). The antiemetics maropitant and ondansetron were also frequently administered to dogs in this study. However, due to the retrospective nature of this study, it was impossible to determine from the records whether the choice to prescribe metoclopramide was primarily for its antiemetic effect, prokinetic effect, or both.

The use of erythromycin in cases with vomiting raises concern that there may be some misconception about the role of prokinetics in the treatment of vomiting. While vomiting may be a clinical sign of GI dysmotility, it may also be due to numerous other mechanisms in hospitalized dogs. Future prospective research is required to determine the occurrence of GI dysmotility in hospitalized dogs with vomiting, and the optimal treatment of dogs with vomiting and GI dysmotility.

In this study, ileus was determined by imaging findings either by the use POCUS or formal abdominal ultrasound. Ileus is typically diagnosed in veterinary medicine through a combination of clinical signs including abdominal distension, decreased or absent bowel sounds, vomiting and/or regurgitation, and diagnostic imaging findings. POCUS is increasingly used in the emergency and critical care setting as a safe and non-invasive means to evaluate canine gastric motility. On average, the stomach and proximal duodenum have four to five sonographically detectable peristaltic contractions per minute, while there are one to three contractions per minute in the jejunum of non-fasted healthy dogs (29). However, the frequency of contractions is reduced with fasting (30). Other methods to assess GI motility are used more commonly for research purposes than clinical purposes given increased invasiveness, cost, and/or impracticality for clinical patients (31). Such methods include scintigraphy, radiographic contrast studies, wireless motility and endoscopy capsules, and gastric emptying breath tests (31–33). Quantification of gastric residual volumes is also useful to assess GI motility, or lack thereof, and is commonly reported in human ICU patients, however were inconsistently documented in this study. A study in healthy cats identified good correlation between scintigraphic and sonographic solid-phase gastric emptying time (34), however to the author’s knowledge a similar relationship has not been demonstrated in dogs. Due to the retrospective nature of this study, the exact criteria that individual clinicians used to diagnose ileus from diagnostic imaging studies was not standardized. Although the sonographically-evident effects of critical illness on GI motility have not been investigated, a normal frequency of contractions is said to rule out generalized ileus (35).

In addition to primary diagnoses associated with GI dysfunction, the majority of the dogs reported herein had one or more other risk factors for dysmotility (1). While the contribution of different risk factors to GI dysmotility in hospitalized dogs is not well characterized in the primary veterinary literature, it has been investigated in human patients and extrapolated to dogs (31, 36). Some of these risk factors become modifiable factors when considering management of dysmotility. Thus while the focus of our study was patterns of prokinetic use with a focus on metoclopramide and erythromycin, the authors advocate a multimodal approach to the treatment of GI dysmotility reflecting the complex and multifactorial pathogenesis. Such a multimodal approach has been reviewed elsewhere including optimizing use of opioids as part of multimodal analgesia, maintenance of euhydration and euvolemia without causing fluid overload, treatment of acid–base and electrolyte disturbances, early enteral feeding, and encouraging patient mobility (1). Additionally, while our study focused on metoclopramide and erythromycin, other prokinetic drugs, such as compounded cisapride, are accessible and have documented efficacy in some studies in dogs (1, 31, 37).

Almost half of the study patient population received proton pump inhibitors (PPI) as an additional GI medication. Though investigation of PPI use was not one of the objectives of this study, it is likely that some of the PPI use in dogs in this study would be considered inappropriate based on guideline recommendations for rational use (38).

The high percentage of French bulldogs in this study is likely due to the high prevalence of regurgitation in brachycephalic dogs (39, 40), the frequency of post-operative regurgitation in dogs undergoing BOAS surgery (41), and evidence supporting the prophylactic use of prokinetics in perianesthetic protocols for brachycephalics (42). Specifically, in a before and after study of a standardized brachycephalic protocol the incidence of postoperative regurgitation in dogs undergoing anesthesia decreased from 35% (14/40) to 9% (4/44) when pre-operative metoclopramide use increased from 13% (5/40) to 89% (39/44) (42). Nonetheless, given that the protocol also included the use of famotidine as an antacid, and changes in opioid use, the effect of the prokinetic cannot be individually determined. Indeed, a more recent study failed to detect a significant reduction in ptyalism or regurgitation with the addition of a metoclopramide CRI to maropitant and pantoprazole in brachycephalic dogs undergoing spinal surgery (43).

While there is limited evidence to support the use of erythromycin to reduce post-operative regurgitation in brachycephalics, it was commonly used for this purpose in the dogs reported in our study. This common usage has also been reported in a prior publication from one of the study institutions (44). In that case series of 29 brachycephalic dogs undergoing a novel hiatal rim repair surgery for persistent regurgitation, two dogs were treated with erythromycin preoperatively, and 21 postoperatively, all in addition to metoclopramide (44). While all dogs were reported to have a good outcome, no data regarding the perceived efficacy of the prokinetics was reported given the confounding of the surgical procedure (44). Further studies are warranted to investigate the role of erythromycin or dual prokinetics to improve GI motility in brachycephalic dogs.

No significant side effects of metoclopramide or erythromycin were identified in the medical record review of cases involved in this study. Nonetheless, our study was not designed to assess drug safety, and it is imperative that clinicians using these medications are aware of the potential for adverse effects. Metoclopramide has been reported to cause adverse effects including mentation and behavior changes such as involuntary spasms, aggression, and hyperactivity to drowsiness (26). Extrapyramidal signs have been described in an English bulldog after a single IV dose of 0.5 mg/kg metoclopramide (45), although it is possible that this dog instead displayed idiopathic head tremors (46). Side effects of erythromycin are considered rare and more likely with high doses, but can include Q-T prolongation, nausea, inappetence, thrombophlebitis after IV injection, and allergic reactions (1, 26). Due to the retrospective nature of this study, it is possible that adverse effects were present but not recognized or documented, and prospective studies will be necessary to better characterize the prevalence of potential side effects.

Erythromycin is a macrolide antibiotic that at doses of 10–25 mg/kg every 8 h is used to treat susceptible infections (26). There is a theoretical concern that use of erythromycin use as a prokinetic could contribute to the development of antibiotic resistance in veterinary medicine. Indeed this concern in human medicine has led some authors to conclude that the use of erythromycin as a prokinetic agent in critically ill patients cannot be recommended unless they have failed other medical management (47). Nonetheless, it is also possible that prokinetic doses of erythromycin are too low to create a selection pressure on enteric bacteria that would favor resistant bacteria, and thus the risk of inducing antimicrobial resistance may be negligible. Prospective studies are necessary to address this knowledge gap in veterinary medicine. Such studies should include meticulous documentation of erythromycin dosing, and serial collection of fecal samples for culture and susceptibility testing to accurately characterize its impact on the development of antibiotic resistance in enteric organisms. Additionally, studies documenting effects of prokinetic erythromycin use on the gastrointestinal microbiome of hospitalized dogs would be valuable. If such studies were to document that use of erythromycin at prokinetic doses did predispose to the development of antimicrobial resistance, then it would be prudent for clinicians to choose other prokinetics as first line agents.

The main limitations of this study were attributed to its retrospective nature. As discussed above, it was possible that adverse effects occurred but were not recorded, so the results may underestimate the potential adverse effects of these prokinetics. Similarly, the retrospective design precludes determination of the effectiveness of the prokinetic medications administered. Although multicenter, the findings of this study may not be representative of the use of prokinetics in hospitalized dogs in other hospitals. Additionally, diagnoses of specific diseases, and of ileus based on POCUS, relied on clinician descriptions in the medical record, and were not standardized. Some reported diagnoses such as “aspiration pneumonia” may be misleading, as without documenting septic inflammation on cytology, histopathology, or a positive bacterial culture, the relative contribution of bacterial infection versus chemical pneumonitis cannot be differentiated. Standardized diagnostic criteria for an imaging diagnosis of ileus, and for underlying diseases affecting enrolled patients, should be used for future prospective studies of prokinetics in dogs. Prospective randomized clinical studies would also benefit from an indicator of illness severity, such as an Acute Patient Physiologic and Laboratory Evaluation Score (APPLE score) (48) when assessing randomization effectiveness and associations between prokinetic use and outcome.

In conclusion, metoclopramide and erythromycin are commonly used in hospitalized dogs, with increasing use of erythromycin alone and dual therapy in the studied institutions. There is currently very limited information on the prevalence of GI dysmotility in hospitalized dogs, relevant risk factors, the effectiveness of metoclopramide and erythromycin, their adverse effects, or long-term outcomes. However, our study demonstrates that these are necessary areas of further research with large scale, multi-institutional prospective studies. In particular, characterization of risk factors, optimal dosing of prokinetics for GI dysmotility, the potential for antimicrobial resistance with erythromycin use as a prokinetic, and further clinical evaluation of other available prokinetics such as cisapride should be investigated.

## Data Availability

The raw data supporting the conclusions of this article will be made available by the authors, without undue reservation.
