# Supplementary material for: Use of erythromycin and metoclopramide in hospitalized dogs: a multicenter historical cohort study
Source: Front Vet Sci. 2025 Apr 25;12:1551312. doi: 10.3389/fvets.2025.1551312 (PMC12063353; doi:10.3389/fvets.2025.1551312)
Supplement: Supplementary file 1 [file Supplementary_file_1.docx]

Randomization Procedure

Cases retrieved by the fee code searches were separated by institution and year into four spreadsheets. The cases in each spreadsheet were sorted chronologically, by date of first prescription of metoclopramide and/or erythromycin. Duplicate entries for patient number were removed from each spreadsheet, leaving a single line for each case treated with metoclopramide and/or erythromycin. For each spreadsheet, the total number of cases was denoted *n*. Subsequently, the positive integers between 1 and *n* were listed in random order by an online research randomization tool (randomizer.org). The randomly ordered list of integers was appended as an additional column alongside the chronological list of cases. The spreadsheets were then sorted by the random numbers, randomizing the order of cases. Cases were sequentially added until 75 cases from each spreadsheet were included in the study.
