# Supplementary material for: Use of erythromycin and metoclopramide in hospitalized dogs: a multicenter historical cohort study
Source: Front Vet Sci. 2025 Apr 25;12:1551312. doi: 10.3389/fvets.2025.1551312 (PMC12063353; doi:10.3389/fvets.2025.1551312)
Supplement: Supplementary file 2 [file Supplementary_file_2.pdf]

Prokinetic retrospective case series

Record ID	__________________________
Centre	◯ TAHMU<br>◯ WAVES
Year	◯ 2018<br>◯ 2023
Patient ID - CStone or Rx	__________________________
First Name	__________________________
Date of birth	__________________________
Date of first prokinetic administration	__________________________
Age (calculated - months)	__________________________
Sex	◯ Male Entire<br>◯ Female Entire<br>◯ Male Neutered<br>◯ Female Speyed

Breed

◯ Mixed
◯ Afghan Hound
◯ Airedale terrier
◯ Alaskan malamute
◯ American Bull Terrier
◯ American Bulldog
◯ Australian Cattle Dog/Blue Heeler
◯ Australian Kelpie
◯ Australian Shepherd
◯ Basset Hound
◯ Beagle
◯ Belgian Malinois
◯ Bernese Mountain dog
◯ Bichon Frise
◯ Blue Heeler
◯ Border Collie
◯ Boxer
◯ British Bulldog
◯ Bull Arab
◯ Bull Terrier
◯ Cairn Terrier
◯ Cavalier King Charles Spaniel
◯ Chihuahua
◯ Cocker Spaniel
◯ Corgi
◯ Coton de Tulear
◯ Daschund
◯ Dalmatian
◯ Doberman
◯ Dogue De Bordeux
◯ English bulldog
◯ English Springer Spaniel
◯ Flat Coated Retriever
◯ Fox Terrier
◯ French Bulldog
◯ German Shephard
◯ German shorthaired pointer
◯ Golden Retriever
◯ Great Dane
◯ Greyhound
◯ Husky
◯ Irish Terrier
◯ Italian greyhound
◯ Jack Russell Terrier
◯ Japanese Spitz
◯ Kelpie
◯ Labrador Retriever
◯ Lagotto
◯ Leonberger
◯ Lhasa Apso
◯ Maltese
◯ Maremma Sheepdog
◯ Mastiff
◯ Miniature Daschund
◯ Miniature Schnauzer
◯ Papillon
◯ Parson Russell Terrier
◯ Pomeranian
◯ Pug
◯ Rhodesian Ridgeback
◯ Rottweiller
◯ Saluki
◯ Samoyed
◯ Shiba Inu
◯ Shih Tzu
◯ Siberian Husky
◯ Staffordshire Bull Terrier
◯ Standard Poodle
◯ Standard Schnauzer

	◯ Toy Poodle<br>◯ West Highland White Terrier<br>◯ Whippet
Recorded bodyweight (kg)	______________________
Clinical Signs /PE findings prior to starting prokinetic<br>(Select where present)	☐ Diarrhea<br>☐ Abdominal pain<br>☐ Inappetence<br>☐ None of the above
What happened to the diarrhoea after starting prokinetic?	☐ Improved<br>☐ Worsened<br>☐ Stayed the same<br>☐ Unclear from the medical record
Did diarrhoea develop after starting the prokinetic	◯ Yes<br>◯ No

Diagnosis	☐ Primary GI (Acute vomiting and/or diarrhoea, no definitive diagnosis)<br>☐ Primary GI (Diaphragmatic hernia and GI entrapment)<br>☐ Primary GI (Infectious)<br>☐ Primary GI (Inflammatory)<br>☐ Primary GI (Food bloat)<br>☐ Primary GI (Foreign body)<br>☐ Primary GI (Gastric dilation and volvulus)<br>☐ Primary GI (Hiatal hernia)<br>☐ Primary GI (intestinal torsion)<br>☐ Primary GI (Neoplasia)<br>☐ Primary GI (Gastroesophageal reflux disease)<br>☐ Snake envenomation<br>☐ Toxicity<br>☐ Extra GI (Endocrine - hypoadrenocorticism, hypothyroidism)<br>☐ Extra GI (Genitourinary)<br>☐ Extra GI (Hepatic)<br>☐ Extra GI (Respiratory)<br>☐ Extra GI (Renal)<br>☐ Extra GI (Thromboembolic disease)<br>☐ Extra GI (Neoplasia)<br>☐ Anaphylaxis<br>☐ Megaesophagus<br>☐ Pancreatitis<br>☐ Esophagitis<br>☐ Sepsis of GI origin<br>☐ Sepsis of non-GI origin (eg. pneumonia)<br>☐ Septic peritonitis<br>☐ Tracheal foreign body<br>☐ Trauma - hbc<br>☐ Neuro - CNS disease<br>☐ Ileus post heat stress/ shock<br>☐ BOAS surgery<br>☐ Retrobulbar abscess<br>☐ Thermal burn(s)<br>☐ Left sided congestive heart failure (LCHF)<br>☐ IMHA<br>☐ Dog fight wound<br>☐ Snake envenomation<br>☐ Vestibular disease from middle ear infection<br>☐ Unclear from medical record
Reason for starting prokinetic	☐ Imaging diagnosis of ileus (FAST, abdominal ultrasound)<br>☐ Clinical signs - regurgitation<br>☐ Clinical signs - vomiting<br>☐ Intolerance of enteral feeding<br>☐ Unknown<br>☐ Prophylactic for BOAS (without history of regurgitation)<br>☐ BOAS with regurgitation history<br>☐ Prophylactic for enteral feeding<br>☐ Prophylactic post abdominal surgery
Select the injectable prokinetic(s) used:	○ Metoclopramide only<br>○ Erythromycin only<br>○ Metoclopramide and erythromcyin
Was a culture obtained within 3 months after commencing injectable erythromycin?	○ Yes<br>○ No

Upload C&S results<br>(if multiple put in zip file and upload)	
Which drug was prescribed first?	◯ Metoclopramide first<br>◯ Erythromycin first<br>◯ Both drugs prescribed at the same time<br>◯ Unable to discern from the medical records
Which department prescribed the first prokinetic?	◯ ECC<br>◯ Medicine<br>◯ Surgery<br>◯ Other<br>◯ Unclear from the medical record
Erythromycin frequency	◯ IV every 6 hours<br>◯ IV every 8 hours<br>◯ Another dosing frequency<br>◯ IV once only
Metoclopramide frequency / dosing	☐ IV every 6 hours<br>☐ IV every 8 hours<br>☐ IV as CRI after an initial bolus<br>☐ IV as CRI without an initial bolus<br>☐ IV once only<br>☐ IV at another dosing frequency
Erythromycin - initial daily dose (mg/kg)	______________________
Erythromycin - MAX daily dose (mg/kg)	______________________
Metoclopramide - initial daily dose (mg/kg)	______________________
Metoclopramide - MAX daily dose (mg/kg)	______________________
Erythromycin - Duration of injectable therapy	◯ less than or equal to 24 hours<br>◯ 24 to 48 hours<br>◯ 48 to 72 hours<br>◯ more than 72 hours
Metoclopramide - Duration of injectable therapy	◯ less than or equal to 24 hours<br>◯ 24 to 48 hours<br>◯ 48 to 72 hours<br>◯ more than 72 hours
Was oral erythromycin given at any time ? (including to go home with)	◯ Yes<br>◯ No
Was oral metoclopramide given at any time ? (including to go home with)	◯ Yes<br>◯ No
Was there any side effects associated with erythromycin/ metoclopramide usage?	◯ Yes<br>◯ No

Describe the side effects and prokinetic used	__________________________________
Risk factors for ileus	☐ SIRS<br>☐ Sepsis<br>☐ Drugs - opioids<br>☐ Ischemia<br>☐ Hypoxia (low SpO2, PaO2, or given O2 supplementation)<br>☐ Abdominal surgery<br>☐ IV fluid bolus (suggestive of hypovolemia)<br>☐ IV fluid rate > maintenance (suggestive of dehydration / ongoing losses)<br>☐ IV KCl or PO K supplementation (suggestive of hypokalemia)<br>☐ IV calcium gluconate supplementation<br>☐ Vasopressor(s)<br>☐ Other - Electrolyte abnormalities<br>☐ Endocrine disease - hypothyroidism<br>☐ General anesthesia<br>☐ Hypotension during general anesthesia (MAP< 65mmHg)<br>☐ Mechanical ventilation for ICU<br>☐ Nil identifiable risk factor<br>☐ Other
Other GI medications?	☐ Maropitant<br>☐ Ondansetron<br>☐ Proton pump inhibitor<br>☐ H2 receptor antagonist (eg. famotidine)<br>☐ Sucralfate<br>☐ Barium (for the purpose of treating GI ulcers)<br>☐ Cisapride<br>☐ Peptobismol<br>☐ Probiotic (eg. prokolin)<br>☐ Other<br>☐ None
Aspiration pneumonia<br>(antimicrobials +/- oxygen therapy)	○ Before prokinetic administration<br>○ After prokinetic administration<br>○ Nil aspiration pneumonia
Was a feeding tube / what feeding tube in place at any time during hospitalisation?<br>(tick all that apply)	☐ NO tube<br>☐ NG tube<br>☐ Oesophagostomy tube<br>☐ PEG / G tube<br>☐ No feeding tube
Where gastric residual volumes reported in the medical record?	○ Yes<br>○ No
Outcome	○ Discharged home<br>○ Discharged to another veterinarian<br>○ Discharged AMA<br>○ Discharged for imminent euthanasia<br>○ Discharged for home trial<br>○ Natural death<br>○ Euthanised
